# The Influence of Transgenic Insect-Resistance and Herbicide-Tolerance Soybean KM2208-23 on the Rhizosphere Micro-Biome

**DOI:** 10.3390/plants15020329

**Published:** 2026-01-21

**Authors:** Xue Song, Xinyao Xia, Shuke Yang, Chaofeng Hao, Hongwei Sun, Fan Li, Xiaohui Xu, Hongxia Zhang, Xingbo Lu

**Affiliations:** 1 The Engineering Research Institute of Agriculture and Forestry, College of Horticulture, Ludong University, 186 Hongqizhong Road, Yantai 264025, China; 2 Shandong Key Laboratory for Green Prevention and Control of Agricultural Pests, Institute of Plant Protection, Shandong Academy of Agricultural Sciences, Jinan 250100, China; 3 Key Laboratory for Safety Assessment (Environment) of Agricultural Genetically Modified Organisms, Ministry of Agriculture and Rural Affairs, Jinan 250100, China

**Keywords:** growth stage, transgenic soybean, microbiome

## Abstract

The consequences of stacking multiple insect-resistance and herbicide-tolerance genes, particularly across the entire plant life cycle, remain inadequately understood. This study investigated the impact of stacked-trait transgenic soybeans on rhizosphere microbial communities across five growth stages (pre-sowing, V3, R3, R5, R8). Using 16S rRNA and ITS sequencing, we compared the rhizosphere microbiome of the transgenic modified soybean (GMO) with its non-transgenic control check (CK). Results showed transient but significant shifts in soil properties (e.g., available nitrogen) and microbial beta diversity during the V3 stages. However, plant developmental stage was the predominant factor shaping microbial succession, with its effect outweighing that of the transgene. No persistent changes in microbial alpha diversity were observed. We conclude that the influence of this stacked-trait soybean on the rhizosphere is growth-stage-specific and represents a minor, recoverable perturbation rather than a sustained ecological impact. These findings contribute to the ecological safety assessment of multi-gene transgenic crops.

## 1. Introduction

The global cultivation area of genetically modified (GM) crops has consistently expanded, with soybean (*Glycine max* L.) being one of the most widely adopted transgenic species [1]. These modifications primarily target agronomic traits such as insect resistance (e.g., via *Bacillus thuringiensis* (Bt) crystal (Cry) and vegetative insecticidal proteins (Vip)) and herbicide tolerance (e.g., to glyphosate or glufosinate), aiming to enhance crop yield and reduce management costs [2]. Notably, stacked-trait varieties, combining multiple transgenes, are increasingly prevalent to address complex field challenges [3]. However, the potential environmental impacts of these transgenic plants, particularly on non-target soil ecosystems, remain a critical component of comprehensive biosafety assessment. Among these concerns, the effects on the structure and function of rhizosphere microbial communities warrant in-depth investigation, given their indispensable roles in soil fertility, nutrient cycling, plant health, and overall ecosystem stability [4].

The rhizosphere, a narrow zone of soil directly influenced by root exudates and activities, harbors an immensely diverse and dynamic microbial consortium [5]. Its community composition is highly sensitive to alterations in plant physiology, biochemistry, and root architecture [6]. Consequently, the introduction of transgenes may indirectly modify the rhizosphere microbiome through changes in root exudation patterns, litter quality, or subtle shifts in plant growth and development [7]. While numerous studies have examined the impact of single-trait GM crops (especially Bt crops) on soil microorganisms, conclusions have been variable, often reporting minor, transient, or plant genotype-dependent effects rather than consistent adverse outcomes attributed solely to the transgene [8,9]. Recent meta-analyses and high-throughput sequencing studies suggest that factors such as plant growth stage, soil type, and agricultural practices often exert stronger influences on microbial communities than the transgenic trait itself [10]. Nevertheless, research on multi-gene stacked traits, particularly those combining insect resistance and novel herbicide tolerance mechanisms, is comparatively limited. The potential synergistic or novel interactive effects of multiple transgenes on the soil microbiome are not yet fully understood, representing a significant knowledge gap.

Most existing temporal studies have focused on limited growth stages, potentially missing critical shifts during key physiological transitions [11]. A comprehensive, stage-resolved analysis—from early seedling establishment through vegetative growth to reproductive maturity and senescence—is essential to capture the full dynamic response of microbial communities to transgenic plants [12]. Furthermore, simultaneous characterization of both bacterial (via 16S rRNA gene sequencing) and fungal (via Internal Transcribed Spacer (ITS) sequencing) communities provides a more holistic view of the rhizosphere ecosystem, as these groups respond differently to plant cues and play distinct ecological roles.

This study aims to explore the potential impact of this complex-trait transgenic soybean on the microbial community in the rhizosphere. We compared the dynamic changes in bacterial (16S rRNA) and fungal (ITS) communities in the rhizosphere of the transgenic lines and their non-transgenic isogenic lines before sowing and at five key growth stages (BQ, V3, R3, R5, and R8). We hypothesized that, given the targeting nature of the exogenous genes and the dominant influence of plant development and environmental factors, any differences between the transgenic and non-transgenic soybean rhizosphere microbial communities would be minor, transient, and within the natural variation range caused by plant growth stages. The results of this study will provide an important scientific basis for the ecological safety assessment of transgenic crops with complex traits.

## 2. Materials and Methods

### 2.1. Genetically Modified Soybean

The transgenic insect-resistance and herbicide-tolerance soybean KM2208-23, which contained the *cry1Ac*, *vip3Aa19*, protoporphyrinogen oxidase gene (*mOsPPO2*), and *pat* genes, was selected as the experimental treatment material, while the non-genetically modified soybean QYZ014 served as the control. The experience was performed at No. 53, Qinglong River Road, Hongshiya Sub-district, Huangdao District, Qingdao City, Shandong Province, at the test base of Qingyuan Compound Co., Ltd. (36°04′37″ N, 120°06′13″ E). Throughout the entire growth period of the soybeans, no herbicides were applied; instead, manual weeding was used as a substitute. This location is characterized by farmland with meadow cinnamon soil. It experiences a temperate continental monsoon climate, with an average yearly temperature of 15 °C, annual precipitation averaging 691 mm, and a frost-free period of 197 days. For each soybean variety, three replicate plots were set up. In each plot, the five-point diagonal sampling method was adopted. Two soybeans were taken from each point. The ten root samples from these five points were combined into one sample and placed in a sampling bag. Two samples were taken from each plot, totaling six replicates for each variety in each period. Rhizosphere soil of GMO and CK soybean at five growth stages including BQ (pre-sowing), V3 (trefoil stage), R3 (beginning pod), R5 (be-ginning seed), and R8 (full maturity) were collected. In brief, the whole root system was pulled out from the field and the loosely attached soil on the roots were removed by shaking. Rhizosphere soil was finally obtained by rinsing the roots with sterile water and high-speed centrifugation of the suspension (Thermo Fisher Scientific Sorvall Legend Micro 21R centrifuge 6000× *g*, Waltham, MA, USA, 20 min).

### 2.2. Determination of Physical and Chemical Properties of Rhizosphere Soil

The total kalium (TK), available kalium (AK), effective nitrogen (EN), pH, organic matter (OM), total phosphorus (TP), available phosphorus (AP), and total nitrogen (TN) of the rhizosphere soil were detected. In brief, the pH of the soil was determined using a pH meter in a suspension with a soil-to-water ratio of 1:2.5. The determination of EN was carried out using 5 g soil sample which was incubated with an alkaline KMnO_4_ solution. The ammonia released during hydrolysis was captured in a boric acid solution and measured by titration with standard sulfuric acid. AP was measured from soil samples which were treated with 0.5 M NaHCO_3_, followed by a reaction with ammonium molybdate and ascorbic acid, and the absorbance was recorded at 880 nm using a UV-8500 spectrophotometer from Tianmei Co., Shanghai, China. AK was isolated using a 1 M ammonium acetate (NH_4_OAc) solution and measured with a flame photometer (M410, Sherwood Scientific Ltd., Cambridge, UK). To quantify TN, fresh samples were extracted with 0.5 M K_2_SO_4_ and analyzed using a TOC analyzer (enviro TOC). OM was determined using the potassium dichromate volumetric method. TP and TK were analyzed using the HF–HClO_4_–HNO_3_ method and an inductively coupled plasma atomic emission spectrometer (iCAP 6300 ICP-OES Spectrometer Thermo Scientific, USA).

### 2.3. DNA Extraction and Amplicon Sequencing

Extract the DNA from 0.5 g rhizosphere soil using the Fast DNA SPIN Kit for Soil (MP Bio-medicals, Solon, OH, USA). Six parallel extractions were performed for each soil sample. DNA quality testing was performed using Qubit^®^ dsDNA HS Assay (Thermo Fisher Scientific, USA) and NanoDrop 2000c (Thermo Fisher Scientific, USA). DNA samples with A260/A280 ratios between 1.8 and 2.0 were used for subsequent experiments. The bacterial 16S rRNA gene was amplified using primers 341F (5′-CCTAYGGGRBGCASCAG-3′)/805R (‘-GACTACHVGGGTATCTAATCC-3′). The fungal ITS region was amplified using primers ITS1F (5′-CTTGGTCATTTAGAGGAAGTAA-3′)/ITS2R (5′-GCTGCGTTCTTCATCGATGC-3′). The amplification reaction contains high-fidelity enzymes (such as KAPA HiFi HotStart ReadyMix), primers (10 μM each 0.2 μL), and template DNA. The amplification procedure is 95 °C 3 min, denaturation: 95 °C 30 s, annealing: 55 °C 30 s (for bacteria) or 50 °C 30 s (for fungi), extension: 72 °C 30 s (30 cycles), final extension: 72 °C 5 min. The library was conducted using the Nextera XT Index Kit (Catalog no. FC-131-1096, Illumina, San Diego, CA,, USA) and the library concentration was quantified using Qubit^®^. Library fragment sizes were confirmed using a Gilent 2100 Bioanalyzer (Santa Clara, CA, USA). After library construction, sequencing was performed using the Illumina NovaSeq PE250 platform (Wekemo Tech Group Co., Ltd., Shenzhen, China).

### 2.4. Bioinformatic Analysis of Amplicon Sequencing Data

The analysis of bacterial 16S rRNA genes and fungal ITS sequences was conducted using QIIME version 1.9 [13] and USEARCH version 10 [14]. Initially, FastQC version 0.11.5 [15] was employed to assess the quality of the reads. Subsequently, Trimmomatic version 0.39 [16] was utilized to trim paired reads with quality scores below Q30. After quality filtering, chimeric sequences were identified and removed using the UCHIME algorithm as implemented in VSEARCH (v2.30.1) in de novo mode. The processed sequences were then clustered, with those exhibiting a similarity greater than 97% being classified as belonging to the same operational taxonomic unit (OTU). Taxonomic classification of the sequences was performed using the SILVA version 138.1 [17] and UNITE version 8.2 databases [18] to distinguish between bacterial and fungal sequences. For α diversity estimation, the OTU tables for bacteria and fungi were standardized using the normalize_table.py script in QIIME, following the cumulative sum scaling (CSS) method. β diversity analysis was conducted using the beta_diversity.py script, also employing the CSS normalization approach. Principal coordinate analysis (PCoA) was performed using the R software environment, version 4.1.0 [19], employing the Vegan package version 2.6.4 [20] and the Tidyverse package version 2.0.0 [21]. PICRUSt2 version 2.6.2 [22] was used to perform functional prediction of 16S rRNA gene sequences in the Kyoto Encyclopedia of Genes and Genomes (KEGG) functional database. Pairwise *T*-test was performed for different groups, with the *p* threshold set at 0.05 (*p* < 0.05 indicates significance). FunGuild version 1.0 [23] was used to perform the ecological function prediction of fungal community. The biomarker bacteria were identified using LEfSe (logarithmic LDA score > 3.0, *p* < 0.05).

## 3. Results

### 3.1. The Influence of GMO on Physical and Chemical Properties of Soil

To investigate the influence of GMO on soil, rhizosphere soil of soybeans from five different growth stages was collected to detect the physical and chemical properties. The five periods were BQ (before sowing), V3 (trefoil stage), R3 (beginning pod), R5 (beginning seed), and R8 (full maturity). Results showed that the EN, OM, and TN indexes showed the significant differences between GMO and CK groups at V3 and R3 periods (Table 1, Wilcoxon rank sum test, *p* < 0.05). The pH index showed the between-group variance at the later stage of soybean growth (R5 and R8). The TK and AP indexes showed the differences at BQ and R3 stages, respectively. In general, genetically modified soybean has a greater impact on the physical and chemical properties of the rhizosphere soil at V3 and R3 stages.

### 3.2. The Basic Information of Amplicon Sequencing Data

At the BQ, V3, R3, R5, and R8 stages, rhizosphere soil of soybean from both the GMO and CK groups was selected for further analysis. We characterized the bacterial and fungal community compositions by sequencing the small ribosomal subunit (16S rRNA) gene fragments and internally transcribed spacer (ITS) sequences, followed by clustering into operational taxonomic units (OTUs) at 97% identity. From 60 samples, we obtained 4,792,307 high-quality 16S rRNA reads and 5,574,493 ITS reads, with individual sample reads ranging from 42,015 to 128,946 and an average of 86,390 reads per sample, representing 3284 bacterial and 821 fungal OTUs. The dilution curve, constructed based on the Shannon index, demonstrated a tendency to plateau, indicating that species richness in this environment does not significantly increase with additional sequencing, thereby satisfying the requirements for subsequent analysis (Figure S1).

### 3.3. The Influence of GMO on Microbial Alpha Diversity of Soybean

**Figure 1:**
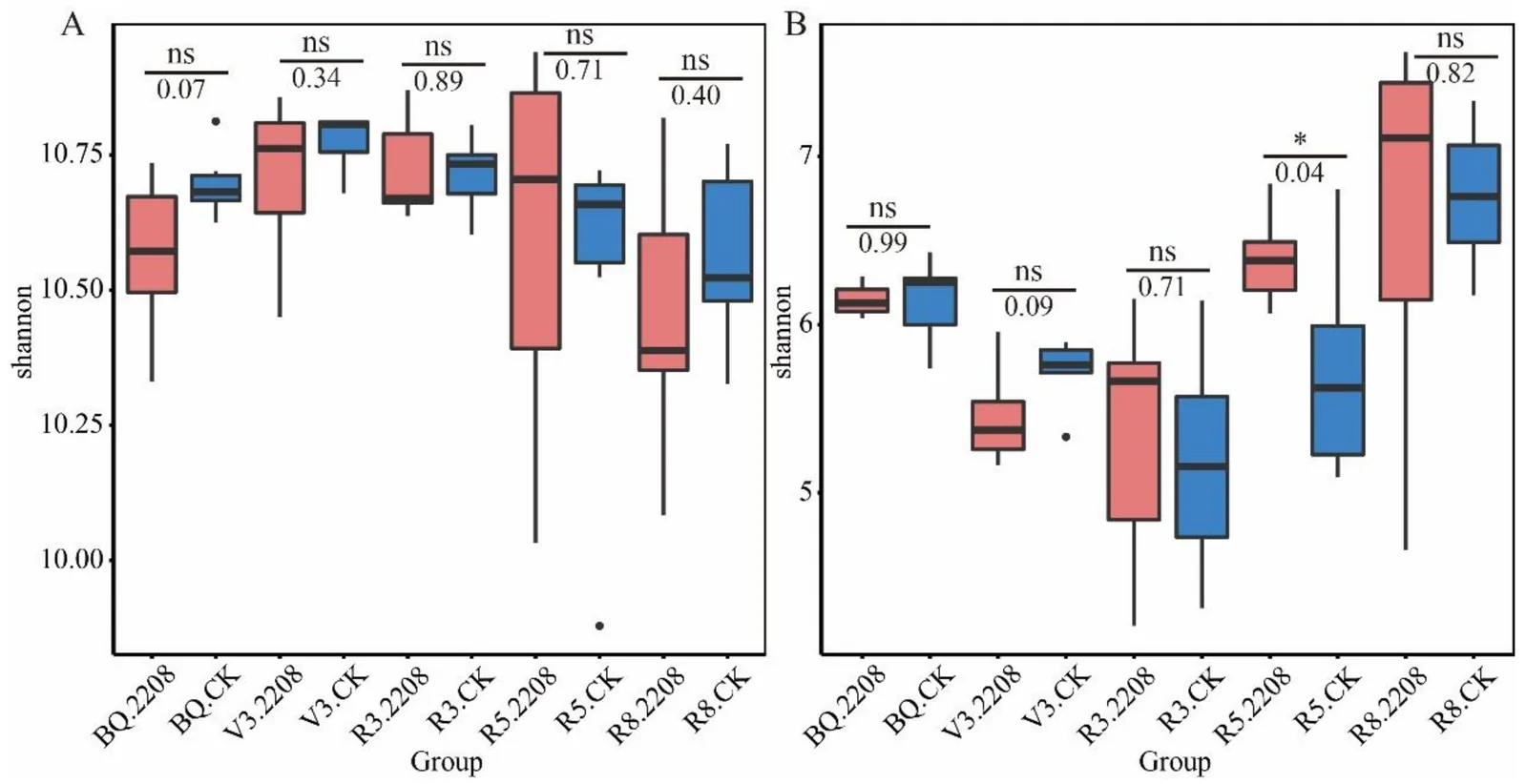
Shannon diversity index (SDI) of bacterial (**A**) and fungal communities (**B**). Along the x-axis, 2208 represented the transgenic soybean KM2208-23. GMO group was marked by red box, CK group was marked by blue box. Wilcoxon rank sum test was used to test for the significant difference between the two groups. “ns” represents the *p*-values > 0.05, which were marked under the transverse lines. * represents the *p*-values < 0.05.

**Figure 2:**
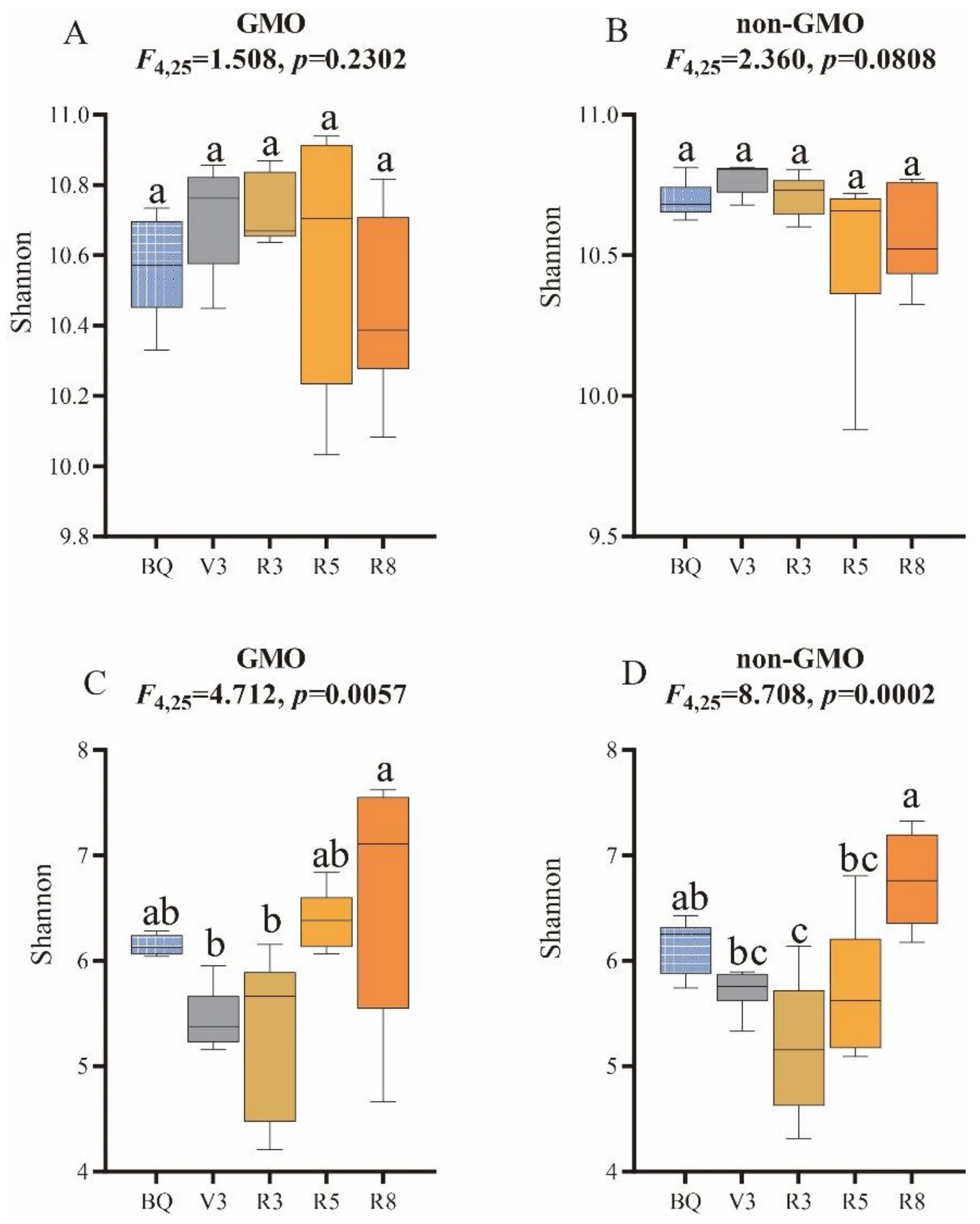
Shannon diversity index (SDI) of bacterial GMO (**A**), CK (**B**), and fungal GMO (**C**), CK (**D**) communities. Different letters indicate significant differences (ordinary one-way ANOVA with Tukey’s multiple comparisons test, *p* < 0.05).

Comparative analyses of Shannon Diversity Index (SDI) were conducted between GMO and CK groups for both bacterial and fungal communities. The SDI for bacterial communities did not exhibit statistically significant differences between GMO and CK groups across the five stages, as determined by the Wilcoxon rank sum test (*p* > 0.05; Figure 1A). In contrast, the SDI for fungal communities was observed to be higher at R5 stage in the GMO groups compared to the CK group (*p* < 0.05; Figure 1B). In addition, the influence of growth stage on microbial alpha diversity was also investigated. As Figure 2A,B show, no significant difference in the bacterial SDI was found across the five stages for GMO or CK groups (Tukey’s multiple comparisons test *p* > 0.05). On the contrary, the SDI of fungal community significantly increased from V3 to R3 stages in both GMO and CK groups (Tukey’s multiple comparisons test *p* < 0.05, Figure 2C,D).

### 3.4. The Influence of GMO on Microbial Beta Diversity of Soybean

**Figure 3:**
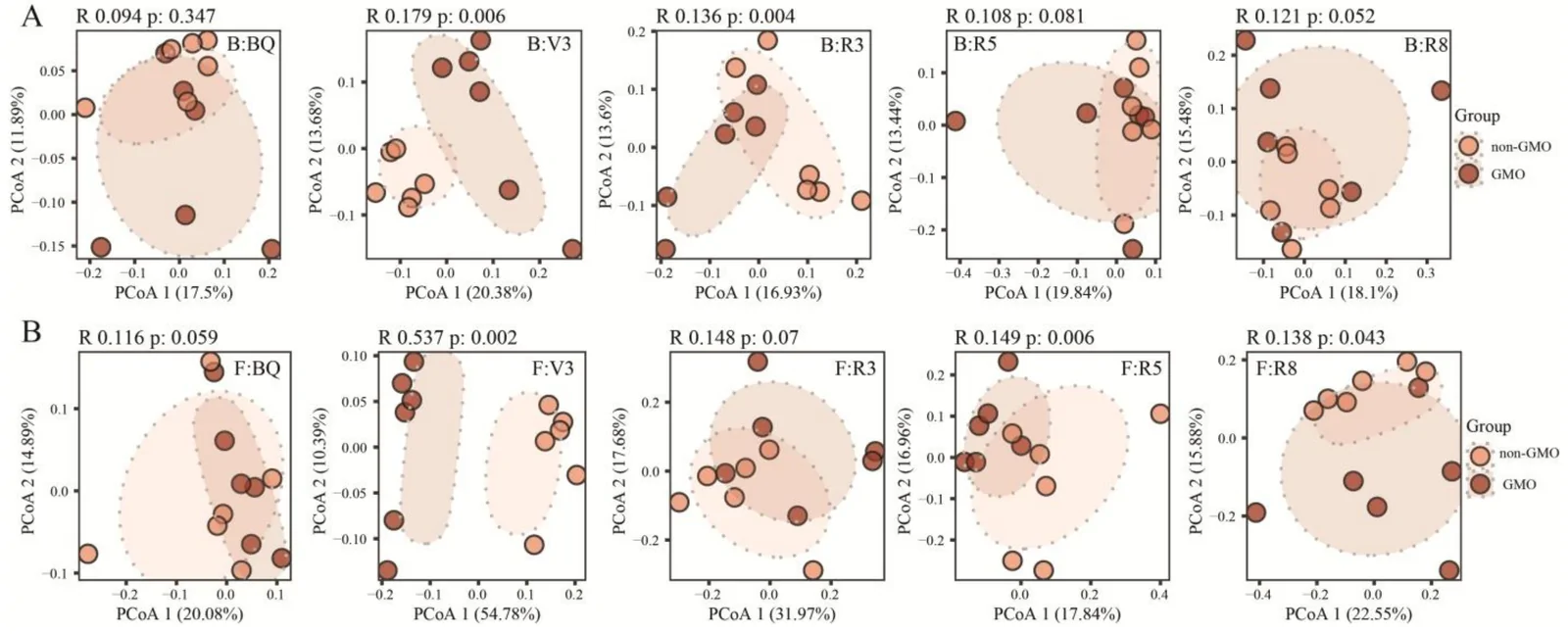
Principal coordinates analysis (PCoA) of bacterial (**A**) and fungal (**B**) communities based on Bray–Curtis distances among the five stages. Significant differences among the groups were determined using PERMANOVA (*n* = 12).

Principal coordinates analysis (PCoA) utilizing Bray–Curtis dissimilarity did not distinguish GMO and CK groups both in bacterial communities (Figure S2A, Adonis R^2^ = 0.022, *p* = 0.097) and fungal communities (Figure S2B, Adonis R^2^ = 0.019, *p* = 0.24) without considering the growth stage. Examining separately the different stages, significant compositional differences in bacterial communities were observed between GMO and CK groups at the V3 (Figure 3A, Adonis R^2^ = 0.179, *p* = 0.006) and R3 (Adonis R^2^ = 0.136, *p* = 0.004) stages along the first principal component. As for fungal community, significant compositional differences were found at V3 (Figure 3B, Adonis R^2^ = 0.537, *p* = 0.002), R5 (Adonis R^2^ = 0.149, *p* = 0.006), and R8 (Adonis R^2^ = 0.138, *p* = 0.043) stages. These results of PCoA indicated that GMO influenced the beta diversity of both bacterial and fungal community at V3 stage.

**Figure 4:**
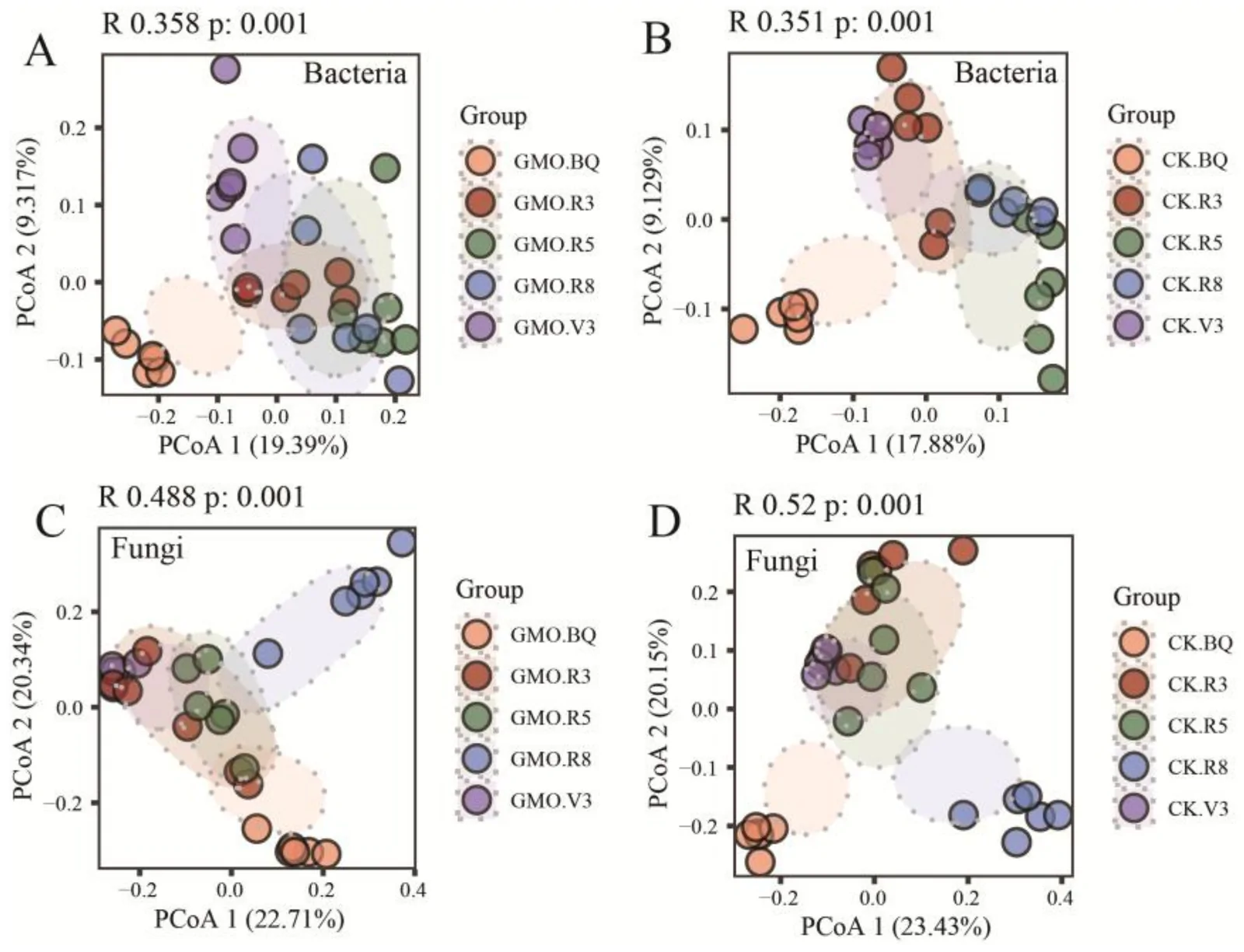
Principal coordinates analysis (PCoA) of bacterial GMO (**A**), bacterial CK (**B**), fungal GMO (**C**), and fungal CK (**D**) communities based on Bray–Curtis distances among the five stages. Significant differences among the groups were determined using PERMANOVA (*n* = 32).

To investigate whether growth stage affects the composition of microbiome communities, PCoA was conducted across the five stages. For bacteria, samples in each growth stage were significantly separated with others for both GMO and CK groups (Figure 4A,B). Fungal communities showed a similar pattern in PCoA results. All these PCoA findings suggest that growth stages deeply influence the composition of microbiome communities, and GMO influence these at special growth stages (Figure 4C,D).

### 3.5. The Influence of GMO on Microbial Community Structure of Soybean

To characterize the microbiota associated with the GMO group, we conducted a comprehensive analysis of the taxonomic compositions of their bacterial and fungal communities. Analysis of microbial communities at the phylum level revealed similar compositions between the GMO and CK groups (Figures S3 and S4). The predominant bacterial phyla (Figure S3A) identified were Proteobacteria (29.81%), Acidobacteriota (18.33%), Actinomycetota (18.32%), and Gemmatimonadota (11.78%). A parallel analysis of fungal communities (Figure S4A) indicated that Ascomycota (68.97%) and Fungi_phy_Incertae_sedis (6.30%) were the dominant phyla. At the genus level, the principal bacterial taxa included *Sphingomonas* (5.43%), *MND1* (2.84%), *Streptomyces* (0.49%), and *Pseudomonas* (0.18%) (Figure S3B). The dominant fungal genera were *Acrophialophora* (6.05%), *Schizothecium* (5.47%), *Alternaria* (1.45%), and *Albifimbria* (1.21%) (Figure S4B). According to the Wilcoxon rank sum test, five bacterial dominant phyla, including Pseudomonadota, Acidobacteriota, Thermoproteota, Chloroflexota, and Myxococcota, showed significant differences between the GMO and CK groups at V3 stage (*p* < 0.05). Similar results were also found in fungal community, in which *Mortierellomycota*, *Mucoromycota*, *Kickxellomycota*, and *Fungi_phy_Incertae_sedis* showed the differences between the two groups at V3 stage. It is worth noting that more fungal genus was found sensitive to GMO than bacteria at V3 stage, that *Acrophialophora*, *Schizothecium*, *Ascomycota_gen_Incertae_sedis*, *Chytridiomycota_gen_Incertae_sedis*, and *Alternaria* were influenced by GMO.

### 3.6. The Influence of GMO on Microbial Biomarkers of Soybean

**Figure 5:**
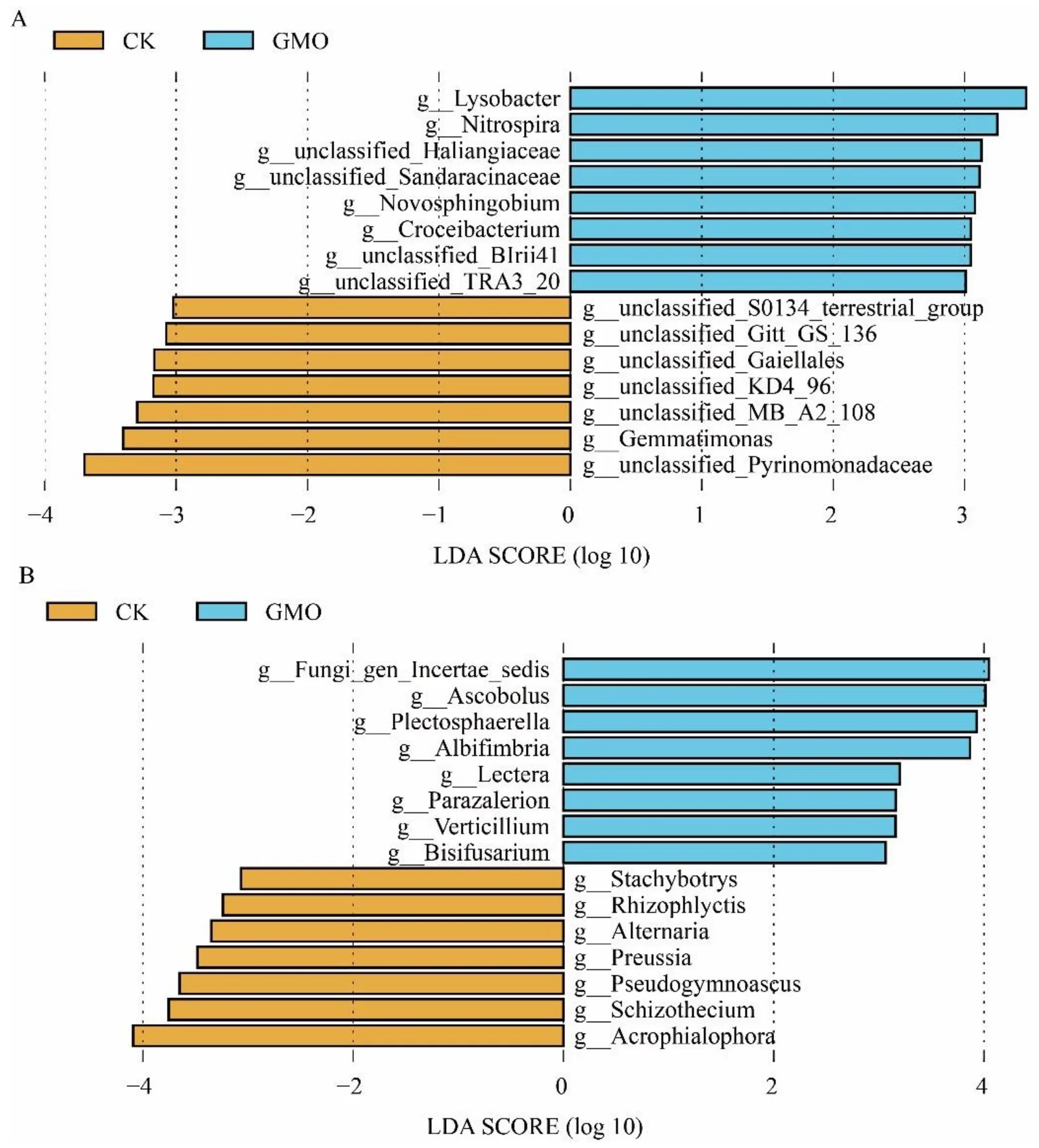
Biomarkers of bacterial (**A**) and fungal (**B**) communities were identified by LEfSe (LDA > 3) between GMO and the CK groups at V3 stage.

Line Discriminant Analysis (LDA) effect size (LEfSe) is an analytical technique that integrates the non-parametric Kruskal–Wallis and Wilcoxon rank sum tests with the effect size derived from LDA. This method is employed to identify biomarkers exhibiting statistically significant differences across various groups. Considering that the bacterial and fungal community of soybean at V3 stage were sensitive to GMO, LDA was performed to find the biomarkers related to GMO at V3 stage. As Figure 5A shows, *Lysobacter*, *Nitrospira*, *Novosphingobium*, and *Croceibacterium* could serve as the bacterial genus-level biomarkers for GMO group. As for fungal community, *Ascobolus*, *Plectosphaerella*, *Albifimbria*, *Lectera*, *Parazalerion*, *Verticillium*, and *Bisifusarium* were the biomarkers for GMO group (Figure 5B).

### 3.7. The Influence of GMO on Microbial Function of Soybean

**Figure 6:**
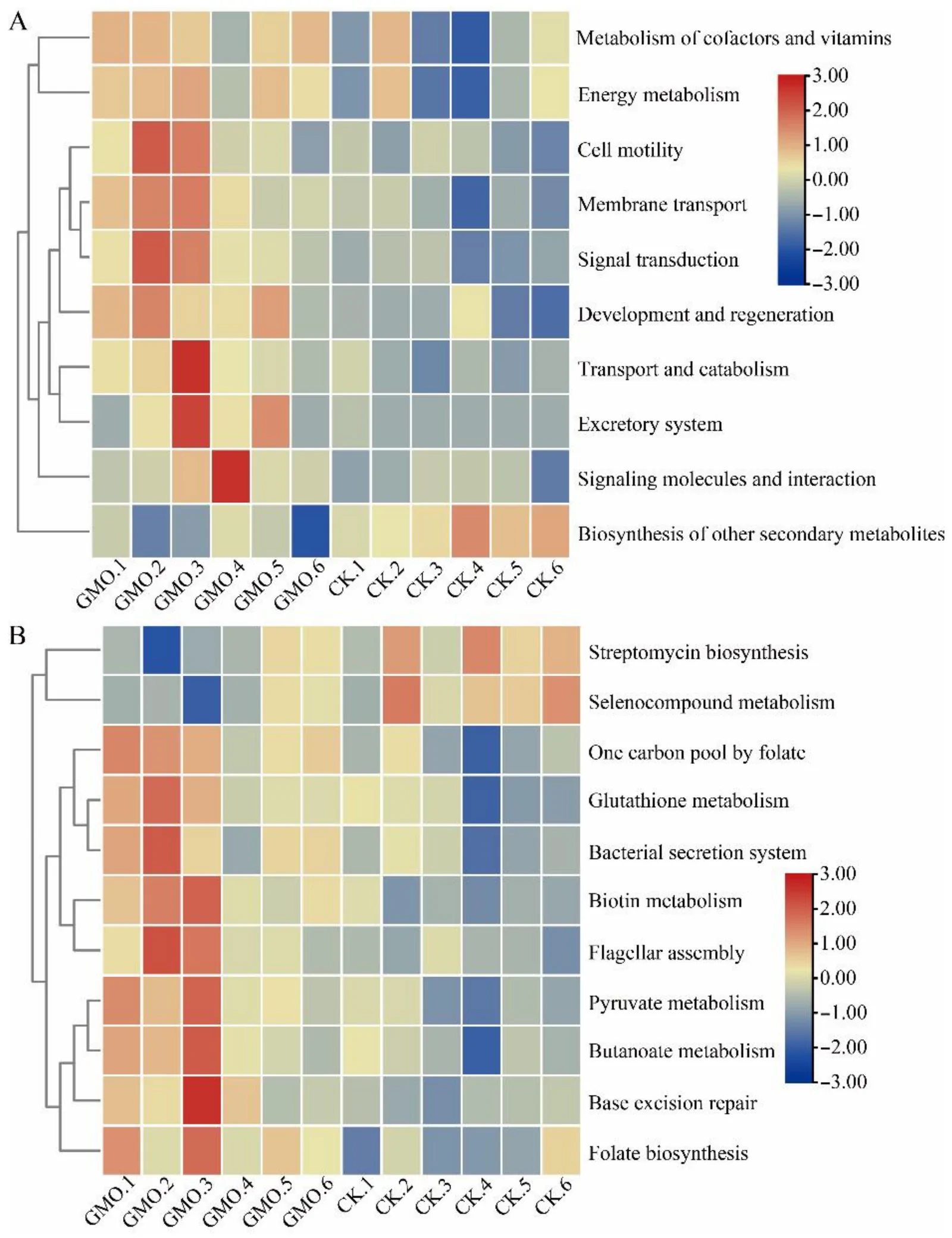
Inter-group KEGG metabolic pathway difference analysis diagram at level 2 (**A**) and level 3 (**B**).

To explore the functional disparities among microbial groups, we employed PICRUSt2 to predict 16S rRNA gene sequences within the KEGG database. Subsequently, we conducted pairwise significance tests between samples using the Wilcoxon rank sum test. Given the rhizosphere microbiome of soybean at V3 stage, our analysis concentrated on the functional differences between the GMO and CK groups at this juncture. As illustrated in Figure 6A, pathways at level 2 related to metabolism of cofactors and vitamins, energy metabolism, cell motility, membrane transport, signal transduction, development and regeneration, transport and catabolism, and energy metabolism were enriched in the GMO group (Figure 6A). Conversely, pathways at level 3 associated with one carbon pool by folate, glutathione metabolism, bacterial secretion system, biotin metabolism, flagellar assembly, pyruvate metabolism, butanoate metabolism, base excision repair, and folate biosynthesis were more prevalent in the GMO group (Figure 6B). In addition, the role that fungi play in the ecosystem was predicted based on fungal taxa using FunGuild. Results showed that, at V3 stage of soybean, endophyte–plant pathogen and wood saprotroph were enriched in GMO group (Figure S5).

## 4. Discussion

This study comprehensively evaluated the temporal impacts of a novel stacked-trait (insect-resistant and herbicide-tolerant) transgenic soybean on the physicochemical properties and microbial communities of the rhizosphere soil. Our multi-stage analysis revealed a nuanced pattern: while the transgenic soybean did induce detectable shifts in specific soil properties and microbiome composition, these effects were largely confined to specific growth stages, most prominently the V3 stages. Furthermore, the influence of the plant’s developmental stage itself exerted a dominant and consistent effect on microbial community assembly, a force that far outweighed the impact of the transgene over the entire growth cycle. These findings align with the growing consensus that the effects of GM crops on soil ecosystems are often subtle, transient, and context-dependent rather than profound and persistent [24,25].

### 4.1. Stage-Specific Modulation of Rhizosphere Environment and Microbiome

The most pronounced differences between GM and non-GM soybean rhizospheres occurred during the V3 and R3 stages. Rather than occurring independently, the observed shifts in soil physicochemical properties and microbial community structure appear to be mechanistically coupled. Significant alterations in soil available nitrogen (EN), organic matter (OM), and total nitrogen (TN) at these stages suggest a potential shift in root exudation patterns or nitrogen uptake efficiency mediated by the transgenic traits. Root exudates are primary drivers of rhizosphere chemistry and microbial recruitment [26]. Modifications in plant physiology due to the expression of cry1Ac, vip3Aa19, mOsPPO2, and pat genes may lead to qualitative or quantitative changes in exudate profiles, particularly during active vegetative growth and reproductive onset, thereby altering nutrient dynamics. This chemical shift likely exerted a selective pressure on the microbiome, which in turn fed back into soil nutrient cycling. For instance, the identification of Nitrospira as a bacterial biomarker for the GM rhizosphere at the V3 stage is directly relevant to the observed fluctuations in soil nitrogen pools. Nitrospira species are key nitrite-oxidizing bacteria involved in the second step of nitrification [26]. Their enrichment suggests that the transgenic roots may have created a microenvironment favoring rapid nitrogen turnover, thereby influencing the levels of available nitrogen (EN) detected in our soil assays. Similarly, the enrichment of Lysobacter, a genus known for high lytic enzyme production and organic matter degradation, correlates with the significant differences in soil organic matter (OM) [27]. This suggests a “plant-driven, microbe-mediated” mechanism where transgenic root exudates stimulate specific functional groups (copiotrophs and nitrogen cyclers), resulting in the transient acceleration of nutrient mineralization observed at the V3 stage [28]. This is supported by the concomitant significant shifts in bacterial and fungal community beta diversity specifically at V3. The identification of specific bacterial (e.g., *Lysobacter*, *Nitrospira*) and fungal (e.g., *Albifimbria*, *Verticillium*) genera as biomarkers for the GM rhizosphere at V3 further underscores this critical stage. The sensitivity of the V3 stage microbiome is notable; it may represent a key window where the plant microbiome is being established and is most receptive to plant-mediated changes.

The lack of persistent divergence in alpha diversity (Shannon index) for bacteria and its only sporadic increase for fungi at R5 indicates that the transgene did not fundamentally reduce or enhance microbial species richness in a lasting manner. This is a crucial finding for ecological risk assessment, as sustained loss of diversity could impair ecosystem resilience. The overwhelming separation of samples by growth stage in PCoA for both treatments powerfully demonstrates that plant phenology is the principal factor structuring the rhizosphere microbiome, consistent with studies on conventional and other GM crops [29]. The transgene effect, while statistically significant at certain points, appears as a secondary modulation within this dominant developmental trajectory.

### 4.2. Functional Implications of Microbial Community Shifts

The predicted profiling (PICRUSt2) at the sensitive V3 stage revealed intriguing functional differences. The enrichment of pathways related to energy metabolism (e.g., pyruvate metabolism, oxidative phosphorylation), membrane transport, and bacterial motility (flagellar assembly) in the GM rhizosphere suggests a potentially more metabolically active or copiotrophic bacterial consortium. This functional prediction corroborates the physicochemical data, supporting the hypothesis that the GM rhizosphere at V3 supports a more active nutrient-cycling community driven by substrate availability. Energy metabolism (such as pyruvate metabolism and oxidative phosphorylation) and membrane transport pathways may be associated with the relative abundance changes in the enriched oligotrophic/nutrient-sensitive bacteria. For instance, Acidobacteriota, which did not appear as a marker in the LEfSe analysis but was significantly different at the phylum level in the differential analysis, showed a significantly lower relative abundance in the GMO group at the V3 stage. Acidobacteria are generally oligotrophic and grow slowly. The decrease in their relative abundance may indicate a shift in the community towards a more copiotrophic state, which typically has a higher basal metabolic rate and nutrient acquisition (membrane transport) activity. This transformation is consistent with the upregulation of “energy metabolism” and “membrane transport” pathways predicted by PICRUSt2. Additionally, certain marker bacterial genera enriched in the GMO group, such as Lysobacter, are known to be beneficial rhizosphere bacteria that produce a variety of hydrolases and actively participate in the carbon and nitrogen cycles. The increase in their abundance may directly contribute to the signaling of pathways such as “cell movement” (such as flagellum assembly) and “bacterial secretion system”, as these functions are closely related to rhizosphere colonization, competition, and interaction [30]. Notably, the enrichment of “bacterial secretion system” pathways might indicate shifts in inter-microbial or plant-microbe interactions, possibly linked to competition or symbiosis [31]. For fungi, the FunGuild prediction indicated a higher relative abundance of endophyte–plant pathogen guilds in the GM rhizosphere at V3. It does not necessarily imply increased disease risk but may reflect a shift in the competitive balance within the fungal community [32]. Some endophytic plant pathogenic fungi are highly responsive to root exudate chemistry [33]. The observed change could be a transient ecological rearrangement rather than a proliferation of pathogens. Long-term field studies are needed to assess the agronomic significance of such compositional shifts.

Although PICRUSt2 and FunGuild provide useful first-level predictions of microbial functional potential, it is important to acknowledge that these approaches rely on reference genomes and ecological guild assignments rather than direct measurements of metabolic activity. Consequently, they may not fully capture strain-level variation, context-dependent gene expression, or metabolite turnover within the rhizosphere environment. For instance, the enrichment of pathways related to energy metabolism, secretion systems, or fungal trophic modes at the V3 stage reflects shifts in predicted genomic potential but does not directly confirm whether these functions were actively expressed or whether they led to measurable biochemical consequences in situ. Therefore, our functional interpretation should be considered inferential rather than determinative. Future work integrating multi-omics approaches—especially rhizosphere metabolomics, metatranscriptomics, isotopic tracing, and targeted quantification of nitrogen- or carbon-related metabolites—will be essential to validate the functional outcomes suggested by predictive models.

### 4.3. Ecological Significance and Biosafety Perspective

From an ecological safety standpoint, our results support the hypothesis that the impact of this stacked-trait transgenic soybean on the rhizosphere microbiome is limited and stage-specific. The coupling of nutrient fluctuations and microbial shifts observed at V3 represents a transient functional response rather than a permanent ecological disruption. The effects were most detectable during peak vegetative/reproductive activity when root exudation is high, and they diminished or became non-detectable at later stages (R5, R8). This “transient perturbation” model is consistent with numerous studies on single-trait Bt crops, where differences often converge with those of conventional crops at maturity [34]. The fact that the core microbial phyla (e.g., Proteobacteria, Acidobacteriota, Ascomycota) remained dominant and unchanged in both treatments reinforces the stability of the overall community structure.

The significant changes in key soil nutrients (EN, TN) at V3 stage, coupled with the microbial community shifts, suggest that the transgenic plant interacts differently with the soil nutrient cycle during these phases. However, these biochemical differences also did not persist, indicating a resilient soil system that buffers short-term changes. The ecological relevance of these temporary shifts is likely minimal, as they fall within the range of variation caused by standard agricultural practices, plant genotype, and environmental fluctuations [35].

## 5. Conclusions

In conclusion, our temporal study demonstrates that the cultivation of the investigated stacked-trait transgenic soybean induces significant but transient and stage-specific alterations in the rhizosphere environment, primarily during the V3 growth stages. These alterations manifest as changes in soil nitrogen parameters, shifts in microbial community composition (particularly for fungi), and modulations in predicted microbial functional potential. Crucially, the overarching influence of soybean developmental stage remains the primary driver of microbial community succession, and no sustained negative impact on microbial diversity was observed. The effects documented here appear to represent minor ecological perturbations rather than fundamental, lasting dysbiosis. These findings contribute to a more nuanced understanding of the interactions between complex transgenic plants and soil ecosystems, supporting the view that their environmental impact is context-dependent and should be evaluated across the entire crop life cycle.

## Figures and Tables

**Table 1:** Changes in the physicochemical properties of the rhizosphere soil of GMO and CK groups at five growth stages.

Indexes	Groups	BQ	V3	R3	R5	R8
TK (g/Kg)	GMO	22.70 ± 1.68 a	19.27 ± 0.54 a	19.77 ± 0.70 a	19.82 ± 1.07 a	19.68 ± 0.26 a
	CK	20.08 ± 0.67 b	20.05 ± 0.64 a	20.43 ± 0.63 a	20.20 ± 0.76 a	19.87 ± 0.58 a
AK (mg/Kg)	GMO	914.00 ± 52.29 a	1012.50 ± 51.72 a	1055.83 ± 58.37 a	1002.00 ± 100.73 a	1013.00 ± 36.3 a
	CK	946.67 ± 41.97 a	1024.83 ± 62.23 a	1056.00 ± 48.43 a	1064.00 ± 55.17 a	996.17 ± 38.97 a
EN (mg/Kg)	GMO	162.00 ± 42.09 a	128.43 ± 21.92 a	95.03 ± 9.29 a	103.33 ± 22.82 a	92.77 ± 16.81 a
	CK	182.33 ± 43.28 a	111.90 ± 15.8 b	114.15 ± 16.8 b	100.75 ± 23.48 a	100.35 ± 18.05 a
pH	GMO	7.35 ± 0.14 a	6.97 ± 0.19 a	7.37 ± 0.27 a	7.00 ± 0.18 b	7.12 ± 0.08 b
	CK	7.42 ± 0.04 a	7.38 ± 0.26 a	7.62 ± 0.15 a	7.62 ± 0.33 a	7.68 ± 0.19 a
OM (g/Kg)	GMO	19.98 ± 0.71 a	17.77 ± 0.69 b	16.85 ± 0.78 b	18.38 ± 0.83 a	20.33 ± 1.66 a
	CK	19.88 ± 0.53 a	18.95 ± 0.32 a	20.28 ± 2.36 a	19.47 ± 1.34 a	19.18 ± 1.99 a
TP (g/Kg)	GMO	0.72 ± 0.31 a	1.32 ± 0.02 a	1.29 ± 0.06 a	1.32 ± 0.1 a	1.33 ± 0.07 a
	CK	1.31 ± 0.04 a	1.26 ± 0.07 a	1.38 ± 0.06 a	1.24 ± 0.11 a	1.26 ± 0.15 a
AP (mg/Kg)	GMO	152.67 ± 7.34 a	150.83 ± 11.63 a	125.00 ± 9.14 b	125.17 ± 14.11 a	130.83 ± 15.16 a
	CK	144.5 ± 11.84 a	137.83 ± 14.41 a	136.17 ± 5.64 a	115.83 ± 17.99 a	118.33 ± 12.27 a
TN (%)	GMO	0.15 ± 0.00 a	0.13 ± 0.00 b	0.12 ± 0.00 b	0.13 ± 0.00 a	0.14 ± 0.01 a
	CK	0.15 ± 0.00 a	0.14 ± 0.00 a	0.14 ± 0.01 a	0.14 ± 0.01 a	0.14 ± 0.01 a

Wilcoxon rank sum test was used to test for the significant difference between the two groups. The same letters indicate that there is no significant difference between the two groups (*p* > 0.05, *n* = 60). BQ, V3, R3, R5, and R8 represent before sowing, trefoil stage, beginning pod, beginning seed, and full maturity stages, respectively. GMO represents transgenic modified soybean group. CK represents control check group. TK, AK, EN, OM, TP, AP, and TN represent total kalium, available kalium, effective nitrogen, organic matter, total phosphorus, available phosphorus, and total nitrogen, respectively.

## Data Availability

Sequencing data in this study can be found in the open repository Zenodo (https://doi.org/10.5281/zenodo.17938657).

## Supplementary Materials

The following supporting information can be downloaded at: https://www.mdpi.com/article/10.3390/plants15020329/s1, Figure S1. Rarefaction curves of 16S (**A**) and ITS (**B**) samples. Different colored curves correspond to different samples. Figure S2. Principal coordinates analysis (PCoA) of bacterial (**A**) and fungal (**B**) communities based on Bray–Curtis distances among the five stages. Significant differences among the groups were determined using PERMANOVA (*n* = 60). Figure S3. Relative abundance of the dominant bacterial phylum (**A**) and genus (**B**) between GMO and CK groups among the 5 stages. Figure S4. Relative abundance of the dominant fungal phylum (**A**) and genus (**B**) between GMO and CK groups among the 5 stages. Figure S5. Metabolic pathway difference analysis diagram of the fungal community between GMO and CK groups.
