# Supplementary material for: The Influence of Transgenic Insect-Resistance and Herbicide-Tolerance Soybean KM2208-23 on the Rhizosphere Micro-Biome
Source: Plants (Basel). 2026 Jan 21;15(2):329. doi: 10.3390/plants15020329 (PMC13462477; doi:10.3390/plants15020329)
Supplement: Supplementary file 1 [file plants-15-00329-s001.zip › plants-4073947-supplementary.pdf]

Supplemental Information

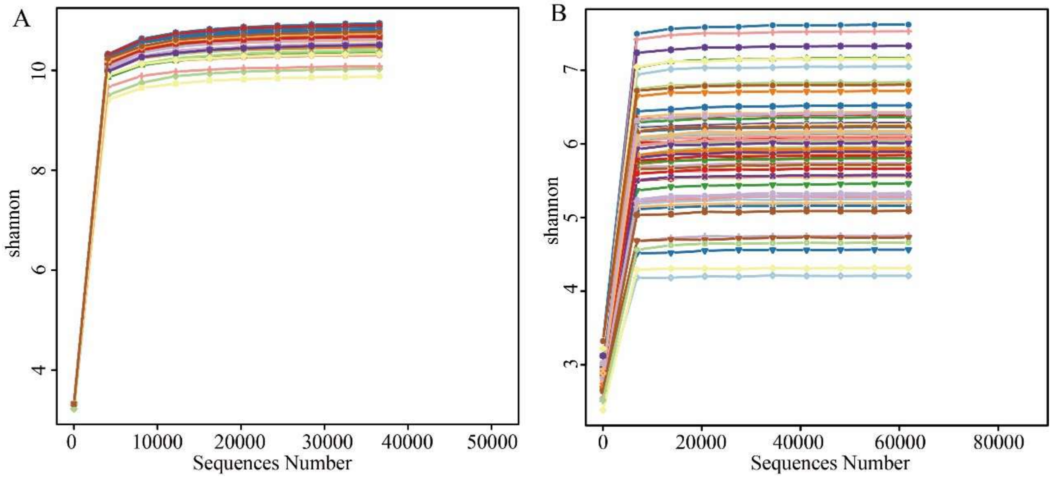

Figure S1. Rarefaction curves of 16S (A) and ITS (B) samples. Different colored curves correspond to different samples.

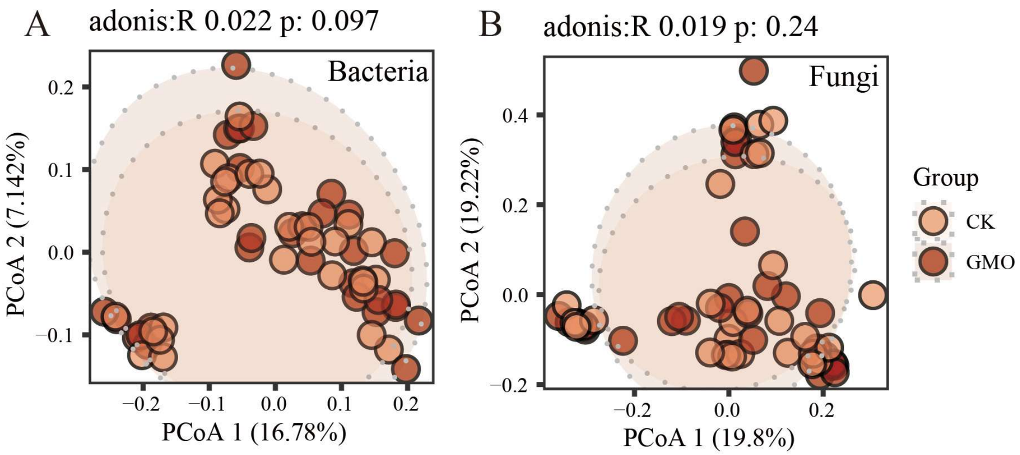

Figure S2. Principal coordinates analysis (PCoA) of bacterial (A) and fungal (B) communities based on Bray–Curtis distances among the five stages. Significant differences among the groups were determined using PERMANOVA (n=60).

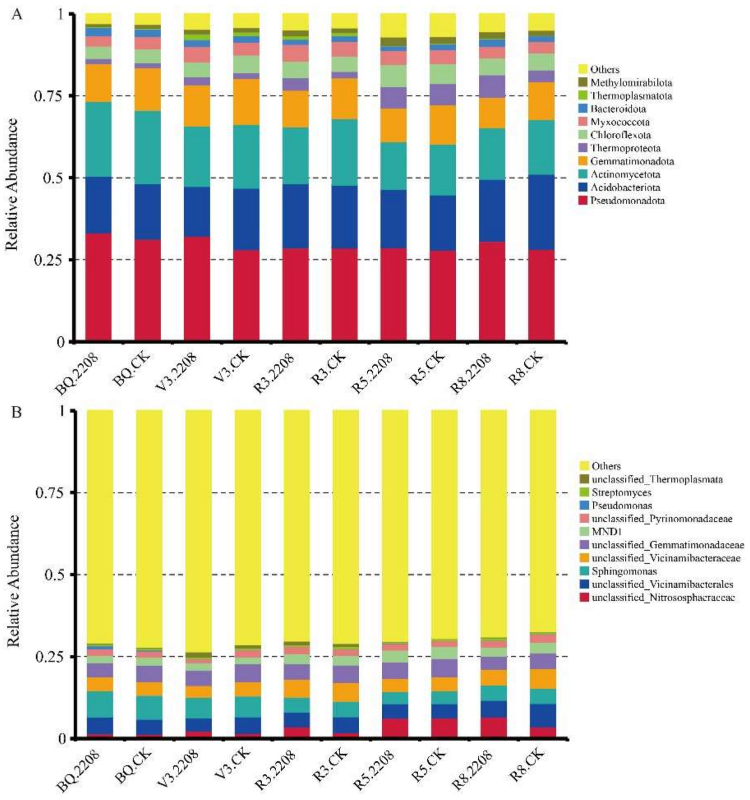

Figure S3. Relative abundance of the dominant bacterial phylum(A) and genus (B) genus between GMO and CK groups among the 5 stages.

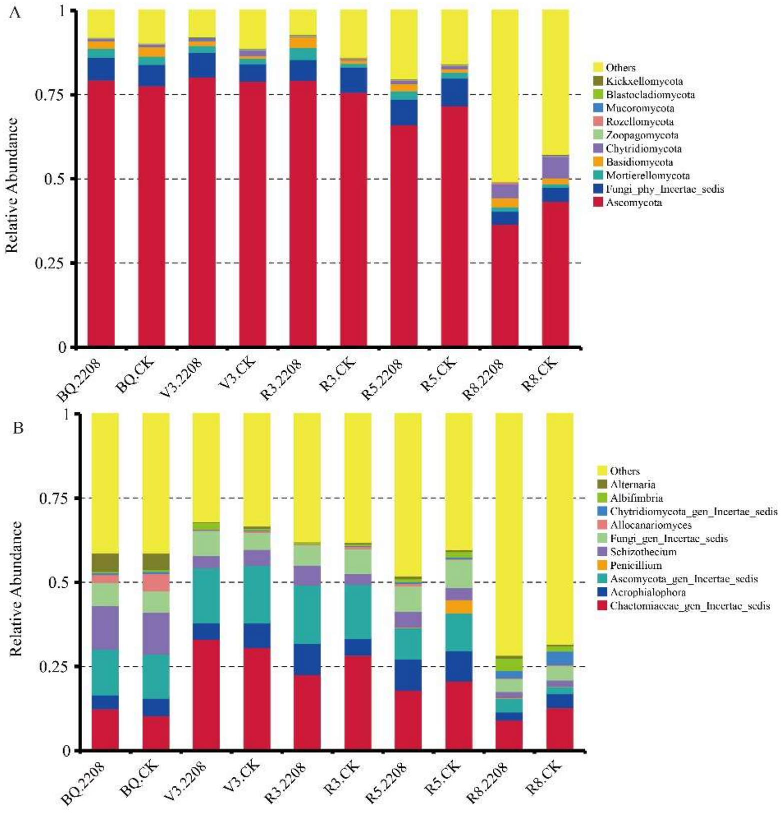

Figure S4. Relative abundance of the dominant fungal phylum(A) and genus (B) genus between GMO and CK groups among the 5 stages.

https://doi.org/10.3390/plants15020329

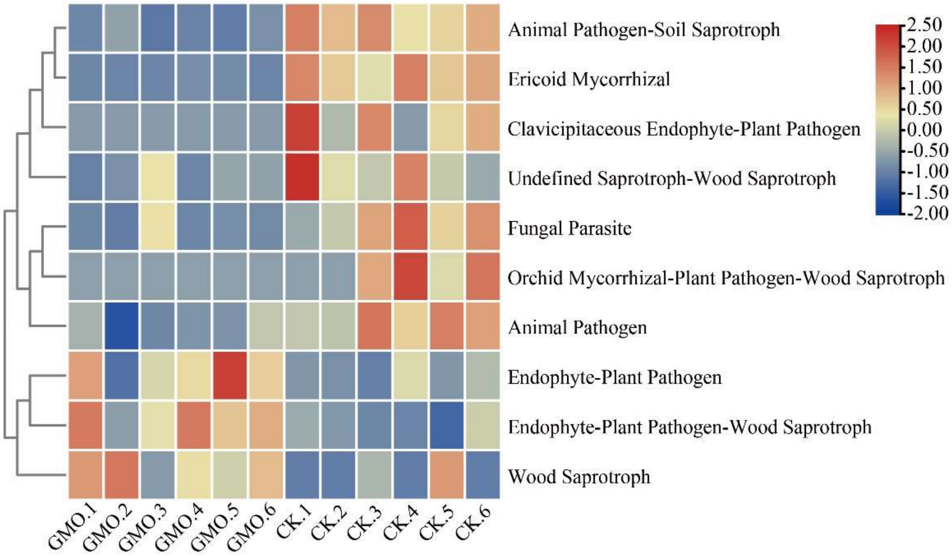

Figure S5. Metabolic pathway difference analysis diagram of the fungal community between GMO and CK groups.
